# Adaptive Capacity of the Habitat Modifying Sea Urchin *Centrostephanus rodgersii* to Ocean Warming and Ocean Acidification: Performance of Early Embryos

**DOI:** 10.1371/journal.pone.0042497

**Published:** 2012-08-03

**Authors:** Shawna A. Foo, Symon A. Dworjanyn, Alistair G. B. Poore, Maria Byrne

**Affiliations:** 1 School of Medical Sciences, The University of Sydney, Sydney, New South Wales, Australia; 2 National Marine Science Centre, Southern Cross University, Coffs Harbour, New South Wales, Australia; 3 Evolution and Ecology Research Centre, School of Biological, Earth and Environmental Sciences, University of New South Wales, Sydney, New South Wales, Australia; 4 Schools of Medical and Biological Sciences, The University of Sydney, Sydney, New South Wales, Australia; National Institute of Water & Atmospheric Research, New Zealand

## Abstract

**Background:**

Predicting effects of rapid climate change on populations depends on measuring the effects of climate stressors on performance, and potential for adaptation. Adaptation to stressful climatic conditions requires heritable genetic variance for stress tolerance present in populations.

**Methodology/Principal Findings:**

We quantified genetic variation in tolerance of early development of the ecologically important sea urchin *Centrostephanus rodgersii* to near-future (2100) ocean conditions projected for the southeast Australian global change hot spot. Multiple dam-sire crosses were used to quantify the interactive effects of warming (+2–4°C) and acidification (−0.3−0.5 pH units) across twenty-seven family lines. Acidification, but not temperature, decreased the percentage of cleavage stage embryos. In contrast, temperature, but not acidification decreased the percentage of gastrulation. Cleavage success in response to both stressors was strongly affected by sire identity. Sire and dam identity significantly affected gastrulation and both interacted with temperature to determine developmental success. Positive genetic correlations for gastrulation indicated that genotypes that did well at lower pH also did well in higher temperatures.

**Conclusions/Significance:**

Significant genotype (sire) by environment interactions for both stressors at gastrulation indicated the presence of heritable variation in thermal tolerance and the ability of embryos to respond to changing environments. The significant influence of dam may be due to maternal provisioning (maternal genotype or environment) and/or offspring genotype. It appears that early development in this ecologically important sea urchin is not constrained in adapting to the multiple stressors of ocean warming and acidification. The presence of tolerant genotypes indicates the potential to adapt to concurrent warming and acidification, contributing to the resilience of *C. rodgersii* in a changing ocean.

## Introduction

Predicting the effects of a rapidly changing climate on natural populations depends not only on measuring the effects of climate stressors on performance and distribution, but also on the likelihood of adaptation to those stressors. The responses of organisms to environmental change include tolerance through acclimatization and phenotypic plasticity, avoidance or shifts in distribution [1], adaptation through genetic change [2] or extinction [2]–[4]. In the short term, phenotypic plasticity may facilitate the persistence of populations [4], but in the long-term, persistence will likely require evolutionary adaptation. Due to the rapid pace of contemporary environmental change compared with the geological past, an understanding of the potential for rapid evolution in response to a changing climate is required to predict evolutionary responses.

Selection by stressful conditions will only result in adaptation if variation in stress tolerance exists within a population, if tolerance of stressors is heritable and if changes in tolerance traits are not constrained by negative genetic correlations with other fitness traits [5], [6]. Empirical data on the evolutionary potential of a wide range of species are needed to determine the potential for adaptation to climatic change [2], [7]. This is particularly true for marine biota for which there is a paucity of data on the potential of populations to genetically adapt to climate-driven change in ocean conditions [8].

The detrimental effects of ocean warming, acidification, hypercapnia and decreased carbonate saturation on marine ecosystems are increasingly well known [8]–[10]. The likelihood of evolutionary responses in natural populations to these stressors may be assessed by two main approaches. Firstly, monitoring genetic changes through time, or contrasting populations under different selection regimes, can provide evidence of selection having resulting in adaptation. Geographically separated populations have been shown to differ in their tolerance to climatic conditions, indicating that past selection has resulted in local adaptation to temperature [11]–[13] and pH [14], [15]. Given dispersal, such population level variation in tolerance can facilitate adaptation to increased temperature and decreased pH at the species level and enhance persistence of the species as the ocean warms and decreases in pH.

Secondly, the potential for adaptation can be assessed through artificial selection or quantitative genetic experiments where individuals of known genotypes are reared in different climatic conditions [2], [3]. This quantitative genetics approach has been used in recent single stressor studies with coral clones and temperature [16] and urchin and mussel larvae with pH [17], and in one multiple stressor study (temperature and acidification) with bryozoan clones [18]. These few marine studies show that the potential to rapidly adapt to one or two stressors varies among species. However, there is still limited knowledge on the presence of heritable (additive genetic) variation for tolerance to climate change stressors.

In this study, the tolerance of early development (to gastrulation) of the sea urchin *Centrostephanus rodgersii* to near-future (2100) ocean change conditions was contrasted among genotypes to assess the potential of this species to adapt to a changing ocean. Previous studies involving echinoderm embryos have shown that gastrulation, is highly sensitive to warming [8], [19]. In echinoderms, very early development (fertilization, cleavage stage embryos) is comparatively robust to increased temperature while post hatching stages (blastula, gastrula) are highly sensitive to warming [8]. We investigated the impact of ocean acidification and ocean warming on development to the gastrula stage in *Centrostephanus rodgersii* in a climate and regionally relevant setting of projected future change for the southeast Australian climate change hot spot [20], [21]. *Centrostephanus rodgersii* is an ecologically important herbivore in rocky reefs where it creates extensive barrens free of foliose macroalgae [22]. This species has expanded its range poleward due to climate driven changes to ocean circulation and warming which has evidently facilitated developmental success and larval migration [1]. Due to the ‘developmental domino effect,’ [8], exposure to stress in early development can result in deleterious downstream effects because the performance of later ontogeny depends on the success of early stages [23].

Here we conducted the first quantitative genetic test involving multiple male × female crosses to assess genetic variation in tolerance to the multiple stressors (pH and temperature) associated with ocean change. The free spawning reproduction of *C. rodgersii* allowed us to create embryos of known parentage and expose each of these to varying temperature and pH conditions [3], [24]. We used the North Carolina II breeding design [24] to quantify the contributions of males, females and genotype × environment (G×E) interactions to variation in performance across nine environments varying in degree of warming (+2–4°C) and acidification (−0.2−0.5 pH units). Interactions between male genotype and environment indicated heritable genetic variation in stress tolerance at the gastrula stage of *C. rodgersii*. Thus there is potential for evolutionary change as a response to selection under changing ocean conditions. We also took the novel step to calculate the genetic correlation of embryo performance across environments showing that adaptation of tolerance to one stressor is not constrained by changes in tolerance to an additional stressor.

## Materials and Methods

### Study Species and Collection Site

*Centrostephanus rodgersii* was collected (3–5 m depth) near Coffs Harbour, New South Wales (30° 15′ S, 153° 08′ E) in July and August 2010 and transferred to large flow through aquaria (3500 L; 20–21°C) shortly after collection. The temperature during the collection period, as indicated by sea surface temperature (SST) recordings during the spawning season, ranges between 20–21°C (http://www.metoc.gov.au/products/data/aussst.php). Permits were obtained for specimen collection (NSW DPI: P10/0023-1.0).

### Spawning and Fertilization

Spawning of *C. rodgersii* was induced by injection of 2–4 ml of 0.5 M KCl. The experiments were conducted in three separate sets of fertilizations or “blocks.” Each block used gametes from separate sets of 3 males and 3 females (see below). All experiments were completed over a 10 day period during the peak reproductive season of *C. rodgersii* [22]. Eggs from each female were placed in separate beakers of fresh, filtered seawater (FSW; 1 µm). Sperm from each male was stored dry at 4°C until use. Egg density was determined in counts of 100 µl aliquots from the egg suspension. Approximately 1000 eggs were placed in rearing containers; 100 ml plastic jars, with mesh sides to allow water flow through. Positioning of the window ensured at least 40 ml of water was in each container at any time as it was constantly renewed. The eggs were supplied with flowing experimental FSW, with randomly assigned temperature/pH conditions for approximately 10 minutes before sperm were introduced. Haemocytometer counts of semen samples were used to determine the amount of sperm required to achieve a consistent egg to sperm ratio across all the blocks. Just prior to fertilization, 1 µl of the semen sample was added to 1 ml of experimental FSW. The amount of diluted sperm to add into each rearing container to achieve the optimal sperm to egg ratio (200∶1; 5×10^3^ sperm/ml, Dworjanyn, unpublished data) was determined from the original sperm count. Before addition of sperm, the flow through system was turned off to allow fertilization and turned back on after 10 minutes to remove excess sperm. The percentage of fertilization in control crosses (ambient temperature-pH) was checked in counts of 50 randomly selected eggs to ensure fertilization rates were acceptable (75–90%), to reduce variation among controls and to avoid problems of polyspermy. Data for one male with low percentage of fertilization in one block were excluded (control and experimental), but this did not change the outcome of the analyses.

### Manipulation of Temperature and pH

The experiments were conducted in a flow through water system with a flow rate of 0.13 ml/sec, ambient pH of 8.06, temperature of 20–21°C, salinity of 35–37 psu, and dissolved oxygen >90%. Experimental treatments were based on model projections for near future (2100) surface ocean waters in the southeast Australia global change hot spot where SST have been warming appreciably for decades [9], [20], [21]. The treatments consisted of three temperatures (control 20.4°C, +2.3°C, +4°C) and three pHNIST levels (control 8.06, −0.26, −0.46 pH units) in all combinations (Table S1).

The experiments were conducted in UV sterilised and filtered (1 µm) FSW that was supplied to three 60 L header tanks. The experimental pH was regulated by injection of pure CO_2_ into two of these tanks using an automatic CO_2_ injection system with two pH controllers (Tunze), set at pH 7.6 and pH 7.8. The CO_2_ was mixed in these tanks using a vortex mixer (Red Sea). A third header tank was allowed to track ambient pH. All header tanks (control and experimental treatment water) were continuously bubbled with air from an aquarium aerator to aid mixing and to maintain dissolved oxygen >90%. A constant volume was maintained in the header tanks using a float valve.

Water from the header tanks was fed into sub-header tanks (20 L) where it was warmed to the required temperatures, +2.3°C and +4°C, using aquarium heaters (200 W, Jager) or un-manipulated for the ambient control. Temperature was automatically regulated using temperature sensors in the rearing containers and a temperature controller (Tunze) connected to the heaters. Water from each sub-header tank was continually circulated using 20 watt pumps to maintain even temperatures within each treatment. Water was delivered individually into individual rearing containers using irrigation dripper valves.

Temperature, pH and salinity were measured daily in all treatments (n = 90 per treatment) using a Hach Hqd Portable Multiprobe. The probe was calibrated frequently using NIST buffers pH 4.0, 7.0 and 10.0 (Oakton) [mean ± SE pH in the three treatments was 8.06±0.006 (control), 7.84±0.0035 (−0.2 units), and 7.64±0.004 (−0.4 units)]. Temperature was also monitored with this meter [mean ± SE temperatures were 20.4±0.31°C (control), 22.7±0.3°C (+2°C) and 24.4±0.3°C (+4°C), see Table S1]. Water samples (100 ml) were also collected daily during the experiment, filtered through a 0.45 µm syringe filter, and fixed with 10 µl of saturated HgCl. These water samples were then used to determine total alkalinity by potentiometric titration using an automatic titrator (Metrohm 888 Titrando) and calibrated against certified reference standards [25]. Experimental *p*CO_2_ (Table S1) was determined from TA, temperature, pHNIST and salinity data using CO2SYS [26] using the dissociation constants of Mehrbach *et al*. [27] as refitted by Dickson and Millero [28].

### The North Carolina II Breeding Design

Single sire-dam crosses were done in three experimental runs (blocks) with each block using gametes from 3 dams and 3 sires crossed in all combinations. Each block thus resulted in 9 full-sib families (total of 27 families). Each family was exposed to each of 9 treatments. Thus each block had a total of 243 containers (3 females × 3 males × 3 pH levels × 3 temperatures × 3 replicates).

At 2 h and 24 h, a random sample of approximately 50 embryos was pipetted from the containers, placed into tubes and fixed with 2% formalin in FSW. The first 30–50 embryos randomly selected from each tube were examined microscopically (Leica) and scored for successful development. At 2 hours, the percentage of cleaving embryos (4 cell stage) was determined. At 24 hours, the percentage of gastrulation was calculated from counts of normal and abnormal gastrulae and arrested embryos. The number of embryos arrested at fertilization (e.g., fertilization envelope only) was low (<1%) indicating that polyspermy was minimal.

### Statistical Analyses

Data on development for each time point were analyzed using analysis of variance (ANOVA) conducted in the PERMANOVA routine of Primer V6 with temperature and pH as fixed factors, experimental block as a random factor, and sire and dam as random factors nested within blocks. Since some significance tests involved quasi F ratios (in which significance tests derived from the F distribution are unreliable [29]), we calculated significance of the F statistics using 9999 permutations of the raw data for all factors [30]. Reaction norms were plotted to visualize the interactions between male genotypes across a range of environments [24]. The genetic correlation of embryo performance (% of normal gastrulae) across temperature and pH environments were used to quantify the G×E interaction using variance components derived from restricted error maximum likelihood (REML) estimates calculated in SYSTAT V10 [31]. REML estimates of variance components for the random factors were calculated in a single analysis with all factors (Temperature, pH, Block, Males, Females). Genetic correlations were calculated using the causal variance components associated with the sire effects (additive genetic (V_A_)) and the interaction effects between sires and each of the environmental factors of temperature (V_AT_), pH (V_A pH_) and both temperature and pH (V_AT pH_). Genetic correlations for the same trait averaged over both types of environments (r^*^_G_), the genetic correlation for the same trait within one environmental class (i.e. temperature; r^*^_G(T)_) and the genetic correlation within the other environmental class (i.e. pH; r^*^_G(pH)_) were calculated using equations from Eisen and Saxton [32]:

r^*^_G_  =  V_A_/(V_A_ + V_AT_ + V_A pH_ + V_AT pH_)

r^*^_G(T)_  =  (V_A_ + V_AT_)/(V_A_ + V_AT_ + V_A pH_ + V_AT pH_)

r^*^_G(pH)_  =  (V_A_ + V_A pH_)/(V_A_ + V_AT_ + V_A pH_ + V_AT pH_)

Among sire variation in 2 hour cleaving embryos is likely to be influenced by the embryo genome and fertilization efficiency, a potential environmental trait of sperm, because zygotic gene expression in sea urchins is switched on before cleavage begins [33]. By gastrulation however, variation is an offspring trait. Therefore genetic correlations were only calculated for the gastrula stage.

## Results

### Cleavage Stage Embryos

**Figure 1:**
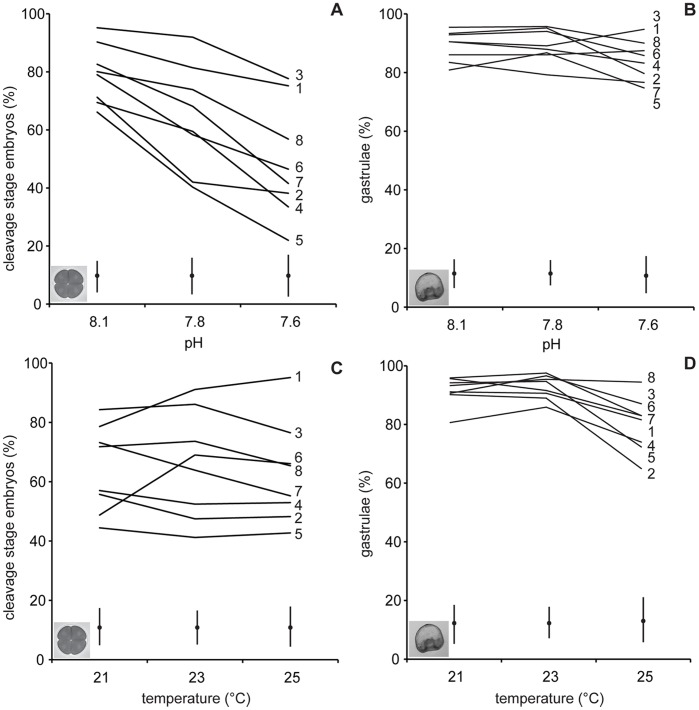
Reaction norms showing the different responses of eight male genotypes to temperature and pH. The reaction norms show percentage of cleavage stage embryos (A,B) and gastrulae (C,D) in experimental temperatures pooled for pH (A,C) and in experimental pH levels pooled for temperature (B,D). Lines represent the mean percentage of paternal half-siblings (n = 8 males). The eight male genotypes and standard errors are indicated.

Decreased pH significantly reduced the percentage of cleavage stage embryos of *Centrostephanus rodgersii* (Table 1, Figure 1A). There was no effect of temperature on cleavage success (Table 1, Figure 1B). There was a significant effect of sire identity on the percentage of cleavage stage embryos across all environments (Table 1). Sire identity contributed the highest percentage (29%) of variance in successful cleavage (Table 1). Maternal identity, by contrast, had no significant effect on cleavage success (Table 1). The percentage of cleavage stage embryos was significantly affected by interactions between temperature and sire, pH and sire, and the three-way interaction among temperature, pH and sire (Table 1) which explained 3%, 3% and 5% of the variation success in cleavage respectively (Table 1).

**Table 1:** Analyses of variance contrasting success in early stage embryos of *Centrostephanus rodgersii* across temperature, pH, male and female identity.

		Cleavage				Gastrulation			
Source	df	MS	F	P	%	MS	F	P	%
Temperature(T)	2	162.3	0.17	0.85	–	10663	11.14	**0.02**	–
pH	2	41357	41.93	**0.001**	–	1173.9	2.50	0.19	–
Block(B)	2	1007.9	0.01	0.99	<0.01	3741.1	0.82	0.59	<0.01
Sire(S) [B]	5	23180	39.99	**<0.001**	29	1165.1	5.90	**0.009**	2
Dam(D) [B]	6	1276.4	2.20	0.13	1	3712.9	18.81	**<0.001**	9
B×T	4	949.1	0.66	0.78	<0.01	965.4	0.52	0.93	<0.01
B×pH	4	980.3	0.76	0.69	<0.01	472.7	1.43	0.21	0.3
T×pH	4	279.4	0.45	0.77	–	358.0	0.84	0.54	–
T×S[B]	10	1370.2	5.34	**0.001**	3	597.2	2.35	**0.04**	2
T×D[B]	12	468.7	1.83	0.11	0.4	1793.8	7.05	**<0.001**	19
pH×S[B]	10	1360.2	2.88	**0.02**	3	350.1	1.15	0.37	<0.01
pH×D[B]	12	543.6	1.15	0.37	0.2	193.0	0.64	0.79	<0.01
S[B]×D[B]	10	580.4	1.36	0.20	0.2	197.4	0.91	0.52	<0.01
B×T×pH	8	614.8	0.77	0.77	<0.01	425.9	1.02	0.45	<0.01
T×pH×S[B]	20	739.4	2.47	**0.009**	5	391.5	1.61	0.10	4
T×pH×D[B]	24	454.4	1.51	0.11	0.7	266.4	1.10	0.39	<0.01
T×S[B]×D[B]	20	255.1	0.60	0.91	<0.01	254.4	1.17	0.28	<0.01
pH×S[B]×D[B]	20	472.0	1.11	0.33	1	303.4	1.40	0.12	0.2
T×pH×S[B]×D[B]	39	299.4	0.70	0.91	<0.01	242.6	1.11	0.30	3
Residuals	409	426.0			57	217.6			60

Temperature (T) and pH were fixed factors, experimental block (B) was a random factor, and male and female identity were random factors nested within block. The percentage of total variance from REML estimates of variance components are shown for random factors. Significant effects are shown in bold.

**Figure 2:**
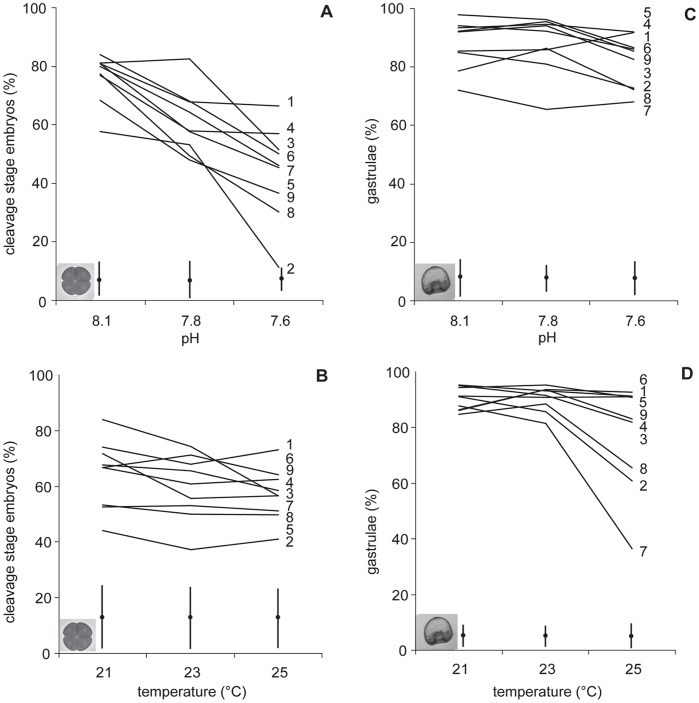
Reaction norms showing the different responses of nine female genotypes to temperature and pH. The reaction norms show percentage of cleavage stage embryos (A,B) and gastrulae (C,D) in experimental temperatures pooled for pH (A,C) and in experimental pH levels pooled for temperature (B,D). Lines represent the mean percentage of maternal half-siblings (n = 9 females). The nine female genotypes and standard errors are indicated.

Reaction norms of the means of paternal half-siblings show the variation among sires in their response to pH and temperature (Figure 1A,B). For pH, there is an overall decrease in cleavage success at low pH with some paternal half siblings being less susceptible to decreased pH than others. At lower pH levels, the variance in the response of cleavage stage embryos increased across families (Figure 1A). Similarly, the reaction norms of maternal half-siblings showed a strong effect of pH that varied among mothers (Figure 2A). The percentage of cleavage stage embryos for both paternal and maternal half siblings was similar across temperature treatments (Figures 1C,2B).

### Gastrulation

Increased temperature, but not decreased pH, significantly reduced the percentage of normal gastrulae (Table 1). Sire and dam identity both affected the percentage of normal gastrulae across all environments, contributing 2% and 9% of the variation, respectively. There were also significant sire and dam by temperature interactions accounting for 2% and 19% of the variation, respectively (Table 1) with a marked drop in gastrulation success at a +4°C warming. The pH by sire and pH by dam interactions were not significant (Table 1). The differences among paternal half siblings in tolerance to acidification and temperature indicated that individual genotypes reacted differently to warming (Figure 1C,D). The differences among maternal half siblings increased with increased warming but not acidification (Figure 2C,D).

There was a positive genetic correlation (r^*^_G_ = 0.28) in the gastrulation trait across all environments indicating that genotypes that performed well in high temperatures also performed well at low pH, and vice versa. There were also positive genetic correlations across the three temperature levels (r^*^_G(t)_ = 0.53) and across the three pH levels (r^*^_G(pH)_ = 0.28). Thus, genotypes that performed well at control temperatures also performed well in higher temperatures, and similarly for pH.

## Discussion

There is substantial evidence that ocean warming and acidification have deleterious effects on a wide range of marine organisms [8], [10], [34], [35] but the evolutionary consequences of this selective pressure are poorly understood. Evolutionary adaptation to climatic stressors will depend on the levels of heritable genetic variation for tolerance to these stressors existing in current populations among other factors such as strength of selection, demographic factors and genetic correlations [2]. Using a quantitative genetic framework aimed at detecting genotype by environment (G×E) interactions in the context of climate change, we found that the tolerance of the progeny of an ecologically important sea urchin, *Centrostephanus rodgersii*, to increased temperature varied significantly among genotypes and that sire effects were important for success in both cleavage and gastrula stages in response to warming and acidification. Maternal identity was important for thermal tolerance in gastrulae. Significant G×E interactions indicates the potential for adaptation to stressors, a feature that would contribute to persistence of this species in a changing ocean.

Across all families, decreased pH, but not increased temperature deleteriously affected early cleavage stage embryos and this was strongly influenced by sire identity with a broad range of success rates among males as shown in the reaction norms. At ambient temperature and pH, fertilization success was high in all control crosses indicating that the sire response to experimental treatments was due to sperm performance, a source of variation noted in other quantitative genetic studies of development in sea urchins and other marine invertebrates [36]. Differences in sire success in free spawning marine invertebrates has been attributed to a range of factors including differences in sperm response to egg pheromones, differences in gamete compatibility and differences in sperm motility [36], [37]. Previous single dam-sire crosses with sea urchins show that decreased pH has deleterious effect on fertilization success [38], [39] in contrast with no deleterious effects on fertilization using gametes from multiple parents, including for *C. rodgersii* [40], [41]. The latter may be due to the paternity success of tolerant sperm from males in mixed semen [36], [42]. The performance of populations of embryos generated from a mixed source of gametes provides an indication of the mean response of *C. rodgersii* fertilization to ocean changes stressors [40], whereas single dam-sire crosses in the present study document the performance of progeny from individual sets of parents.

The strong contribution of sire identity to the variance in cleavage success is consistent with studies showing that differences in sperm performance between individual male sea urchins drive variation in fertilization rates [36], [43], [44]. Non-additive genetic differences in male-female compatibility driven by the sperm bindin-egg bindin receptor system [43] may be exacerbated by stress. Variation in paternity success may also have a genetic component as zygotic gene expression in sea urchin development is switched on soon after fertilization [33]. The basis for variation in paternity success in *C. rodgersii* families is likely to result from mechanisms (genetic and environmental) that would not operate in isolation.

The low proportion of variance attributed to female identity with respect to differences in cleavage was not expected because variation in early cleavage success is likely to be influenced by maternal transcripts (e.g. maternal mRNAs) and variation in egg quality (phenotypic plasticity, e.g. egg nutrients) [33], [45], [47]. The lack of an effect of increased temperature (+2–4°C) on very early development in *C. rodgersii* is similar to that documented for other sea urchin species [8]. This is suggested to be due to the presence of protective maternal factors (e.g., cell defense proteins) in the eggs that buffer the effects of stress on early embryos [45], [46]. Our results indicated that maternal protection in cleaving embryos did not vary significantly among the mothers assayed. The extent of maternal influence (e.g. mRNAs) wanes as the zygotic genome takes over control of development [33], [46].

At the gastrula stage, increased temperature, but not decreased pH, reduced embryo performance, with a marked decrease in success of gastrulation in some families with 4°C warming, similar to that found for another sea urchin species [8], [48]. Gastrulation in echinoderm embryos is sensitive to increased temperature [8], [40]. Sire and dam identity both affected gastrulation success in *C. rodgersii* with a strong interaction between temperature and parent. The influence of male indicates a genotypic effect while the influence of female indicates a combination of environmental and genetic effects. Maternal effects in echinoderms are strongly related to variation in egg cytoplasmic contents (as above), which may be due to genotype (e.g. stress gene expression) or phenotypic plasticity associated with maternal history and diet during egg development [49]. The importance of these factors varies among life history stages [36], [47], [50], [51]. We found no evidence of interactions between male and female genotypes for either cleavage or gastrulation trait and thus no evidence of non-additive genetic variance that could result from differences in egg and sperm compatibility.

A recent quantitative genetic study involving the effects of the single stress of ocean acidification on sea urchin development found that female identity strongly influenced the size of *Strongylocentrotus franciscanus* larvae, with negligible influence of sire [17]. Individual females produced larger larvae, regardless of pH treatment. The consistent influence of mother on larval size across all pH levels indicate that egg (nutritive) provisioning does not interact with pH, but contributes additively to that effect. The findings of Sunday *et al.* [17] for *S. franciscanus* are consistent with our results for *C. rodgersii*. For both species, female identity influenced outcome (*S. franciscanus*: larval size, pH; *C. rodgersii*: gastrula, thermal tolerance).

Temperature is a major factor controlling early development in echinoderms and therefore ocean warming is likely to be the most important contemporary ocean change stressor, especially in areas with a high rate of warming. A 4°C warming exceeds tolerance limits for many echinoderms across latitudes [8], [13], [48], [52]. It is well known that thermal tolerance varies among individuals and populations for many terrestrial species [53], but there are only a few studies that have contrasted the performance of genotypes of marine species from a single population across differing climatic conditions (temperature and/or pH) [16]–[18]. Our study appears to be the first to use a sire by dam breeding design to quantify the genetic versus environmental sources of variation in tolerance to the two major ocean change stressors, warming and acidification, in the context of near future projections. The variation in tolerance to the climatic stressors seen here for *C. rodgersii* is consistent with recent quantitative genetic studies on the potential for adaptation to other anthropogenic stressors facing marine organisms. The tolerance of amphipods to metal contamination of their diets [54] and of polychaetes to water-borne metals [55] both varied significantly among genotypes. Pistevos *et al.* (2011) used a clone × temperature × pH design with the bryozoan *Celleporella hyalina* and demonstrated the existence of genetic variation in growth and reproduction traits. The few studies to date that have used quantitative genetic designs that replicate genotypes (clones, full-sib or half-sib families) across ocean acidification and/or warming conditions are outlined in Table S2.

Understanding differences in performance of genotypes across environments of varying stress (i.e., G×E interactions) is essential for predicting how tolerance to environmental stressors may evolve [3]. The interaction between male identity and temperature for gastrulation in *C. rodgersii* provides evidence of genotypic variation in tolerance to thermal stress. The progeny of some males were strongly affected by warmer temperatures while others were relatively unaffected (visualized by the reaction norms). Selection mediated by increasing temperature would thus be expected to favor tolerant genotypes from this population in future ocean change scenarios.

While the presence of genetic variation in tolerance to a single stressor indicates the potential for directional selection to result in more tolerant populations, such adaptation may be constrained by the genetic relationships among traits within a single environment, and among traits expressed across multiple environments [31], [55]. Evolving tolerance to one stressor may be costly, and negative genetic correlations may result in trade-offs between traits (e.g., a high performing genotype in one environment doing poorly in another) [55]. Trade-offs underlie important theories of life history and evolutionary specialization, and have the potential to confound predictions from experimental studies that examine only one environmental stressor [8], [55], [56]. We found no evidence for such trade-offs, with positive genetic correlations between embryo performance expressed in multiple environments. For gastrulation there were positive genetic correlations among three levels of pH, among three levels of temperature, and across all combinations of both stressors. These indicate that genotypes tolerant to increased temperature were also tolerant to decreased pH, suggesting that evolution of greater tolerance to either stressor is not likely to be constrained by trade-offs in performance across environments. Longer-term rearing experiments are required to test whether thermal or pH tolerance in *C. rodgersii* may result in reductions in other fitness parameters (e.g., larval performance, metamorphosis, reproductive output).

The presence of a G×E interaction, the potential for interaction between ocean warming and acidification [57], [58], and the potential for trade-offs between tolerance traits and other fitness traits emphasize the need for ocean change studies to include multiple stressors and consider the genotypes of organisms being tested. Sea urchins provided a model to quantify the importance of G×E interactions on development. Our results add to the emerging body of evidence that responses of marine organisms to the stressors associated with climate change will vary within species; among clones [16], [18], among maternal half-siblings [17] among life history stages [8], and among populations [11], [13], [59]. To understand this variability, quantitative genetics provides the framework to discern between environmentally induced plastic responses and microevolutionary adaptation [3].

In conclusion, embryonic development in *C. rodgersii* as seen in performance of gastrulae is particularly vulnerable to increased temperature. Both male and female parents influenced the effects of acidification and warming, and the G×E interactions indicated the differential tolerance to temperature among genotypes. The presence of tolerant genotypes, and the lack of a trade-off between tolerance to pH and tolerance to warming contribute to the potential of *C. rodgersii* to adapt to concurrent ocean warming and acidification, adding to the resilience of this ecologically important species in a changing ocean. It will be important to determine the interactive effects of warming and acidification on larval growth in families of *C. rodgersii.* This will determine the potential that moderate warming might alleviate the stunting effects of acidification on larvae [8], [57], [60] and if genotype and maternal effects influence this.

## Supporting Information

Table S1 **Experimental conditions in experiments with** ***Centrostephanus rodgersii*** **embryos.** Mean values are shown for experimental treatments with standard error in brackets. 20.4°C/pH 8.06 were the mean parameters for the control treatment FSW. *p*CO_2_ was calculated in CO2SYS using data on total alkalinity determined for three water samples (TA = 2272.61, SE = 17.77, n = 3) and mean water conditions at the level of the rearing containers (n = 90 per treatment). Also provided are the temperature and pH ranges for each experimental block.
(DOC)

Table S2 **Studies with marine animals and plants that use breeding designs as applied here to** ***Centrostephanus rodgersii*** **to test for within-population genetic variation in tolerance to climate change stressors (temperature and/or acidification).** Studies were found using a systematic literature search in ISI Web of Science using the search term ((climat* or warming or acidification) and (marine or ocean) and (‘quantitative genetics’ or heritabil* or ‘genotype by environment’ or genetic)). Each study uses experimental designs that replicate genotypes (clones, full-sib or half-sib families) across environmental conditions and are thus able to detect an interaction between genotype and the stressor indicative of genetic variation in stress tolerance, or estimate the heritability of stress tolerance. The table lists the species, the stressor, the experimental design and the traits measured.
(DOC)
