# Supplementary material for: Adaptive Capacity of the Habitat Modifying Sea Urchin Centrostephanus rodgersii to Ocean Warming and Ocean Acidification: Performance of Early Embryos
Source: PLoS One. 2012 Aug 3;7(8):e42497. doi: 10.1371/journal.pone.0042497 (PMC3411790; doi:10.1371/journal.pone.0042497)
Supplement: Table S1 — Experimental conditions in experiments with Centrostephanus rodgersii embryos. Mean values are shown for experimental treatments with standard error in brackets. 20.4°C/pH 8.06 were the mean parameters for the control treatment FSW. pCO2 was calculated in CO2SYS using data on total alkalinity determined for three water samples (TA = 2272.61, SE = 17.77, n = 3) and mean water conditions at the level of the rearing containers (n = 90 per treatment). Also provided are the temperature and pH ranges for each experimental block. (DOC) [file pone.0042497.s001.doc]

**SUPPORTING INFORMATION**

**Table S1. Experimental conditions in experiments with *Centrostephanus rodgersii* embryos.** Mean values are shown for experimental treatments with standard error in brackets. 20.4°C/pH 8.06 were the mean parameters for the control treatment FSW. *p*CO_2_ was calculated in CO2SYS using data on total alkalinity determined for three water samples (TA = 2272.61, SE = 17.77, n = 3) and mean water conditions at the level of the rearing containers (n = 90 per treatment). Also provided are the temperature and pH ranges for each experimental block.

| **pH** | | 8.06 (0.006) | | | 7.84 (0.0035) | | | | 7.64 (0.004) | | |
| --- | --- | --- | --- | --- | --- | --- | --- | --- | --- | --- | --- |
| **Temperature (°C)** | | 20.4 (0.31) | 22.7 (0.3) | 24.4 (0.3) | | 20.4 (0.31) | 22.7 (0.3) | 24.4 (0.3) | 20.4 (0.31) | 22.7 (0.3) | 24.4 (0.3) |
| ***p*_CO2 (matm)_** | | 530.3 | 537.9 | 543.3 | 1043.4 | | 1063.6 | 1078.4 | 1716.4 | 1754 | 1781.7 |
| **Block 1**  **range** | **pH** | 8.002 – 8.068 | | | 7.814 – 7.866 | | | | 7.602 – 7.710 | | |
|  | **Temp** | 21.7-21.8 | 23.26-24.04 | 24.78-25.98 | 21.7-21.8 | | 23.26-24.04 | 24.78-25.98 | 21.7-21.8 | 23.26-24.04 | 24.78-25.98 |
| **Block 2 range** | **pH** | 8.036-8.01 | | | 7.798-7.88 | | | | 7.6-7.662 | | |
|  | **Temp** | 20.28-21.32 | 22-23.12 | 24.22-25.28 | 20.28-21.32 | | 22-23.12 | 24.22-25.28 | 20.28-21.32 | 22-23.12 | 24.22-25.28 |
| **Block 3 range** | **pH** | 8.128-8.138 | | | 7.814-7.848 | | | | 7.662-7.674 | | |
|  | **Temp** | 20.02-20.18 | 22.38-22.64 | 23.96-23.98 | 20.02-20.18 | | 22.38-22.64 | 23.96-23.98 | 20.02-20.18 | 22.38-22.64 | 23.96-23.98 |
