# Supplementary material for: Adaptive Capacity of the Habitat Modifying Sea Urchin Centrostephanus rodgersii to Ocean Warming and Ocean Acidification: Performance of Early Embryos
Source: PLoS One. 2012 Aug 3;7(8):e42497. doi: 10.1371/journal.pone.0042497 (PMC3411790; doi:10.1371/journal.pone.0042497)
Supplement: Table S2 — Studies with marine animals and plants that use breeding designs as applied here to Centrostephanus rodgersii to test for within-population genetic variation in tolerance to climate change stressors (temperature and/or acidification). Studies were found using a systematic literature search in ISI Web of Science using the search term ((climat* or warming or acidification) and (marine or ocean) and (‘quantitative genetics’ or heritabil* or ‘genotype by environment’ or genetic)). Each study uses experimental designs that replicate genotypes (clones, full-sib or half-sib families) across environmental conditions and are thus able to detect an interaction between genotype and the stressor indicative of genetic variation in stress tolerance, or estimate the heritability of stress tolerance. The table lists the species, the stressor, the experimental design and the traits measured. (DOC) [file pone.0042497.s002.doc]

**SUPPORTING INFORMATION**

**Table S2. Studies with marine animals and plants that use breeding designs as applied here to *Centrostephanus rodgersii* to test for within-population genetic variation in tolerance to climate change stressors (temperature and/or acidification).** Studies were found using a systematic literature search in ISI Web of Science using the search term ((climat* or warming or acidification) and (marine or ocean) and (‘quantitative genetics’ or heritabil* or ‘genotype by environment’ or genetic)). Each study uses experimental designs that replicate genotypes (clones, full-sib or half-sib families) across environmental conditions and are thus able to detect an interaction between genotype and the stressor indicative of genetic variation in stress tolerance, or estimate the heritability of stress tolerance. The table lists the species, the stressor, the experimental design and the traits measured.

| **Species** | **Stressor** | **Experimental design** | **Traits** |
| --- | --- | --- | --- |
| *Acropora millepora* (coral) symbiont [1] | temperature | among clonal lineages | photosynthesis, gene expression, growth* |
| [2] | temperature | full sib families x temperature | settlement rates, mitochondrial metabolism, gene expression |
| [3] | temperature/light | full sib families x temperature/light | fluorescence, larval settlement |
| *Celleporella hyalina.* (bryozoan) [4] | temperature, pH | clones x temperature x pH | growth, reproduction |
| *Centrostephanus rodgersii* (urchin) [5] | temperature, pH | male x female x temperature x pH | early development |
| *Rhizophora mangle* (mangrove) [6] | hydrological stress related to sea level change | female genotype x environment | survival, growth |
| *Saccostrea glomerata* (oyster) [7] | pH | full sib families x pH | growth |
| *Strongylocentrotus franciscanus* (urchin) [8] | pH | male x female x pH | growth |
| *Zostera marina* (seagrass) [9] | temperature | clone diversity x temperature | growth |

1 Czásár NBM, Ralph PJ, Frankham R, Berkelmans R, van Oppen MJH (2010) Estimating the potential for adaptation of corals to climate warming. PLoS ONE 5: e9751.

2 Meyer E, Davies S, Wang S, Willis BL, Abrego D, Juenger TE, Matz MV (2009) Genetic variation in responses to a settlement cue and elevated temperature in the reef-building coral *Acropora millepora*. [Mar Ecol Prog Ser](http://www.int-res.com/journals/meps/meps-home/) [392:](http://www.int-res.com/abstracts/meps/v392/) 81-92.

3 [Kenkel](http://rspb.royalsocietypublishing.org/search?author1=C.+D.+Kenkel&sortspec=date&submit=Submit) D, [Traylor](http://rspb.royalsocietypublishing.org/search?author1=M.+R.+Traylor&sortspec=date&submit=Submit) MR, [Wiedenmann](http://rspb.royalsocietypublishing.org/search?author1=J.+Wiedenmann&sortspec=date&submit=Submit) J[A, Salih](http://rspb.royalsocietypublishing.org/search?author1=A.+Salih&sortspec=date&submit=Submit) A, Matz MV (2011) Fluorescence of coral larvae predicts their settlement response to crustose coralline algae and reflects stress. Proc R Soc B 278: 2691-2697.

4 Pistevos JCA, Calosi P, Widdicombe S, Bishop JDS (2011) Will variation among genetic individuals influence species responses to global climate change? Oikos 120: 675-689.

5 Present study

6 Proffitt CE, Travis SE (2010) Red mangrove seedling survival, growth, and reproduction: effects of environment and maternal genotype. Est Coast [33:](http://www.springerlink.com/content/1559-2723/33/4/) 890-901

7 Parker LM, Ross PM, O’Connor WA (2011) Populations of the Sydney rock oyster, *Saccostrea glomerata*, vary in response to ocean acidification. Mar Biol 158: 689-697.

8 Sunday JM, Crim RN, Harley CDG, Hart MW (2011) Quantifying rates of evolutionary adaptation in response to ocean acidification. PLoS ONE 6: e22881.

9 Ehlers A, Worm B, Reusch TBH (2008) Importance of genetic diversity in eelgrass *Zostera marina* for its resilience to global warming. [Mar Ecol Prog Ser](http://www.int-res.com/journals/meps/meps-home/) 355: 1-7.
